# A First-Order Logic with Frames

**DOI:** 10.1007/978-3-030-44914-8_19

**Published:** 2020-04-18

**Authors:** Adithya Murali, Lucas Peña, Christof Löding, P. Madhusudan

**Affiliations:** ETH Zurich, Zurich, Switzerland; 9 University of Illinois at Urbana-Champaign, Department of Computer Science, Urbana, IL USA; 10 RWTH Aachen University, Department of Computer Science, Aachen, Germany

**Keywords:** Program Verification, Program Logics, Heap Verification, First-Order Logic, First-Order Logic with Recursive Definitions

## Abstract

We propose a novel logic, called *Frame Logic* (FL), that extends first-order logic (with recursive definitions) using a construct $\textit{Sp}(\cdot )$ that captures the *implicit supports* of formulas— the precise subset of the universe upon which their meaning depends. Using such supports, we formulate proof rules that facilitate frame reasoning elegantly when the underlying model undergoes change. We show that the logic is expressive by capturing several data-structures and also exhibit a translation from a *precise* fragment of separation logic to frame logic. Finally, we design a program logic based on frame logic for reasoning with programs that dynamically update heaps that facilitates local specifications and frame reasoning. This program logic consists of both localized proof rules as well as rules that derive the weakest tightest preconditions in FL.

### 1 Introduction

Program logics for expressing and reasoning with programs that dynamically manipulate heaps is an active area of research. The research on separation logic has argued convincingly that it is highly desirable to have *localized logics* that talk about small states (heaplets rather than the global heap), and the ability to do *frame reasoning*. Separation logic achieves this objective by having a tight heaplet semantics and using special operators, primarily a separating conjunction operator $*$ and a separating implication operator (the magic wand $-*$).

In this paper, we ask a fundamental question: can classical logics (such as FOL and FOL with recursive definitions) be extended to support localized specifications and frame reasoning? Can we utilize classical logics for reasoning effectively with programs that dynamically manipulate heaps, with the aid of local specifications and frame reasoning?

The primary contribution of this paper is to endow a classical logic, namely first-order logic with recursive definitions (with least fixpoint semantics) with frames and frame reasoning.

[⋆] Equal contribution [✉] Corresponding Author

A formula in first-order logic with recursive definitions (FO-RD) can be naturally associated with a *support*— the subset of the universe that determines its truth. By using a more careful syntax such as guarded quantification (which continue to have a classical interpretation), we can in fact write specifications in FO-RD that have very precise supports. For example, we can write the property that $x$ points to a linked list using a formula $list(x)$ written purely in FO-RD so that its support is precisely the locations constituting the linked list.

In this paper, we define an extension of FO-RD, called Frame Logic (FL) where we allow a new operator $Sp(\alpha)$ which, for an FO-RD formula $\alpha$, evaluates to the support of $\alpha$. Logical formulas thus have access to supports and can use it to *separate* supports and do frame reasoning. For instance, the logic can now express that two lists are disjoint by asserting that $Sp(list(x)) \cap Sp(list(y)) = \emptyset$. It can then reason that in such a program heap configuration, if the program manipulates only the locations in $Sp(list(y))$, then $list(x)$ would continue to be true, using simple frame reasoning.

The addition of the support operator to FO-RD yields a very natural logic for expressing specifications. First, formulas in FO-RD have the same meaning when viewed as FL formulae. For example, $f(x) = y$ (written in FO-RD as well as in FL) is true in any model that has $x$ mapped by $f$ to $y$, instead of a specialized "tight heaplet semantics" that demands that $f$ be a partial function with the domain only consisting of the location $x$. The fact that the support of this formula contains only the location $x$ is important, of course, but is made accessible using the support operator, i.e., $Sp(f(x) = y)$ gives the set containing the sole element interpreted for $x$. Second, properties of supports can be naturally expressed using set operations. To state that the lists pointed to by $x$ and $y$ are disjoint, we don't need special operators (such as the $*$ operator in separation logic) but can express this as $Sp(list(x)) \cap Sp(list(y)) = \emptyset$. Third, when used to annotate programs, pre/post specifications for programs written in FL can be made *implicitly* local by interpreting their supports to be the localized heaplets accessed and modified by programs, yielding frame reasoning akin to program logics that use separation logic. Finally, as we show in this paper, the weakest precondition of specifications across basic loop-free paths can be expressed in FL, making it an expressive logic for reasoning with programs. Separation logic, on the other hand, introduces the magic wand operator $-*$ (which is inherently higher-order) in order to add enough expressiveness to be closed under weakest preconditions [38].

We define frame logic (FL) as an extension of FO with recursive definitions (FO-RD) that operates over a multi-sorted universe, with a particular foreground sort (used to model locations on the heap on which pointers can mutate) and several background sorts that are defined using separate theories. Supports for formulas are defined with respect to the foreground sort only. A special background sort of *sets* of elements of the foreground sort is assumed and is used to model the supports for formulas. For any formula $\varphi$ in the logic, we have a special construct $Sp(\varphi)$ that captures its support, a set of locations in the foreground sort, that intuitively corresponds to the precise subdomain of functions the value of $\varphi$ depends on. We then prove a *frame theorem* (Theorem 1) that says that changing a model $M$ by changing the interpretation of functions that are not in the support of $\varphi$ will not affect the truth of the formula $\varphi$. This theorem then directly supports frame reasoning; if a model satisfies $\varphi$ and the model is changed so that the changes made are disjoint from the support of $\varphi$, then $\varphi$ will continue to hold. We also show that FL formulae can be translated to vanilla FO-RD logic (without support operators); in other words, the semantics for the support of a formula can be captured in FO-RD itself. Consequently, we can use any FO-RD reasoning mechanism (proof systems [19, 20] or heuristic algorithms such as the natural proof techniques [24, 32, 37, 41]) to reason with FL formulas.

We illustrate our logic using several examples drawn from program verification; we show how to express various data-structure definitions and the elements they contain and various measures for them using FL formulas (e.g., linked lists, sorted lists, list segments, binary search trees, AVL trees, lengths of lists, heights of trees, set of keys stored in the data-structure, etc.)

While the sensibilities of our logic are definitely inspired by separation logic, there are some fundamental differences beyond the fact that our logic extends the syntax and semantics of classical logics with a special support operator and avoids operators such as $*$ and $-*$. In separation logic, there can be many supports of a formula (also called heaplets)— a heaplet for a formula is one that *supports its truth*. For example, a formula of the form $\alpha \vee \beta$ can have a heaplet that supports the truth of $\alpha$ or one that supports the truth of $\beta$. However, the philosophy that we follow in our design is to have a *single* support that supports the truth value of a formula, whether it be *true or false*. Consequently, the support of the formula $\alpha \vee \beta$ is the *union* of the supports of the formulas $\alpha$ and $\beta$.

The above design choice of the support being *determined* by the formula has several consequences that lead to a deviation from separation logic. For instance, the support of the negation of a formula $\varphi$ is the same as the support of $\varphi$. And the support of the formula $f(x) = y$ and its negation are the same, namely the singleton location interpreted for $x$. In separation logic, the corresponding formula will have the same heaplet but its negation will include *all* other heaplets. The choice of having determined supports or heaplets is not new, and there have been several variants and sublogics of separation logics that have been explored. For example, the logic DRYAD [32, 37] is a separation logic that insists on determined heaplets to support automated reasoning, and the *precise* fragment of separation logic studied in the literature [29] defines a sublogic that has (essentially) determined heaplets. The second main contribution in this paper is to show that this fragment of separation logic (with slight changes for technical reasons) can be translated to frame logic, such that the unique heaplet that satisfies a precise separation logic formula is its support of the corresponding formula in frame logic.

The third main contribution of this paper is a program logic based on frame logic for a simple while-programming language destructively updating heaps. We present two kinds of proof rules for reasoning with such programs annotated with pre- and post-conditions written in frame logic. The first set of rules are local rules that axiomatically define the semantics of the program, using the smallest supports for each command. We also give a frame rule that allows arguing preservation of properties whose supports are disjoint from the heaplet modified by a program. These rules are similar to analogous rules in separation logic. The second class of rules work to give a *weakest tightest precondition* for any postcondition with respect to non-recursive programs. In separation logic, the corresponding rules for weakest preconditions are often expressed using separating implication (the magic-wand operator). Given a small change made to the heap and a postcondition $\beta$, the formula $\alpha -\!\!* \beta$ captures all heaplets $H$ where if a heaplet that satisfies $\alpha$ is joined with $H$, then $\beta$ holds. When $\alpha$ describes the change effected by the program, $\alpha -\!\!* \beta$ captures, essentially, the weakest precondition. However, the magic wand is a very powerful operator that calls for quantifications over heaplets and submodels, and hence involves second order quantification. In our logic, we show that we can capture the weakest precondition with only first-order quantification, and hence first-order frame logic is closed under weakest preconditions across non-recursive programs blocks. This means that when inductive loop invariants are given also in FL, reasoning with programs reduces to reasoning with FL. By translating FL to pure FO-RD formulas, we can use FO-RD reasoning techniques to reason with FL, and hence programs.

In summary, the contributions of this paper are:

- A logic, called *frame logic* (FL) that extends FO-RD with a support operator and supports frame reasoning. We illustrate FL with specifications of various data-structures. We show a translation to equivalent formulas in FO-RD.
- A program logic and proof system based on FL including local rules and rules for computing the weakest tightest precondition. FL reasoning required for proving programs is hence reducible to reasoning with FO-RD.
- A separation logic fragment that can generate only precise formulas, and a translation from this logic to equivalent FL formulas.

The paper is organized as follows. Section 2 sets up first-order logics with recursive definitions (FO-RD), with a special uninterpreted foreground sort of locations and several background sorts/theories. Section 3 introduces Frame Logic (FL), its syntax, its semantics which includes a discussion of design choices for supports, proves the frame theorem for FL, shows a reduction of FL to FO-RD, and illustrates the logic by defining several data-structures and their properties using FL. Section 4 develops a program logic based on FL, illustrating them with proofs of verification of programs. Section 5 introduces a precise fragment of separation logic and shows its translation to FL. Section 6 discusses comparisons of FL to separation logic, and some existing first-order techniques that can be used to reason with FL. Section 7 compares our work with the research literature and Section 8 has concluding remarks.

### 2 Background: First-Order Logic with Recursive Definitions and Uninterpreted Combinations of Theories

The base logic upon which we build frame logic is a first order logic with recursive definitions (FO-RD), where we allow a foreground sort and several background sorts, each with their individual theories (like arithmetic, sets, arrays, etc.). The foreground sort and functions involving the foreground sort are *uninterpreted* (not constrained by theories). This hence can be seen as an uninterpreted combination of theories over disjoint domains. This logic has been defined and used to model heap verification before [23].

We will build frame logic over such a framework where supports are modeled as subsets of elements of the foreground sort. When modeling heaps in program verification using logic, the foreground sort will be used to model *locations of the heap*, uninterpreted functions from the foreground sort to foreground sort will be used to model *pointers*, and uninterpreted functions from the foreground sort to the background sort will model *data fields*. Consequently, supports will be subsets of locations of the heap, which is appropriate as these are the domains of pointers that change when a program updates a heap.

We define a signature as $\Sigma = (S; C; F; \mathcal{R}; \mathcal{I})$, where $S$ is a finite non-empty set of sorts. $C$ is a set of constant symbols, where each $c \in C$ has some sort $\tau \in S$. $F$ is a set of function symbols, where each function $f \in F$ has a type of the form $\tau_1 \times \ldots \times \tau_m \to \tau$ for some $m$, with $\tau_i, \tau \in S$. The sets $\mathcal{R}$ and $\mathcal{I}$ are (disjoint) sets of relation symbols, where each relation $R \in \mathcal{R} \cup \mathcal{I}$ has a type of the form $\tau_1 \times \ldots \times \tau_m$. The set $\mathcal{I}$ contains those relation symbols for which the corresponding relations are inductively defined using formulas (details are given below), while those in $\mathcal{R}$ are given by the model.

We assume that the set of sorts contains a designated "foreground sort" denoted by $\sigma_\mathsf{f}$. All the other sorts in $S$ are called background sorts, and for each such background sort $\sigma$ we allow the constant symbols of type $\sigma$, function symbols that have type $\sigma^n \to \sigma$ for some $n$, and relation symbols have type $\sigma^m$ for some $m$, to be constrained using an arbitrary theory $T_\sigma$.

A formula in first-order logic with recursive definitions (FO-RD) over such a signature is of the form $(\mathcal{D}, \alpha)$, where $\mathcal{D}$ is a set of recursive definitions of the form $R(\boldsymbol{x}) := \rho_R(\boldsymbol{x})$, where $R \in \mathcal{I}$ and $\rho_R(\boldsymbol{x})$ is a first-order logic formula, in which the relation symbols from $\mathcal{I}$ occur only positively. $\alpha$ is also a first-order logic formula over the signature. We assume $\mathcal{D}$ has at most one definition for any inductively defined relation, and that the formulas $\rho_R$ and $\alpha$ use only inductive relations defined in $\mathcal{D}$.

The semantics of a formula is standard; the semantics of inductively defined relations are defined to be the least fixpoint that satisfies the relational equations, and the semantics of $\alpha$ is the standard one defined using these semantics for relations. We do not formally define the semantics, but we will formally define the semantics of frame logic (discussed in the next section and whose semantics is defined in the Technical Report [25]) which is an extension of FO-RD.

## 3 Frame Logic

We now define Frame Logic (FL), the central contribution of this paper.

FL formulas: $\varphi ::= t_\tau = t_\tau \mid R(t_{\tau_1}, \ldots, t_{\tau_m}) \mid \varphi \wedge \varphi \mid \neg\varphi \mid ite(\gamma : \varphi, \varphi) \mid \exists y : \gamma.\ \varphi$
$\tau \in S$, $R \in \mathcal{R} \cup \mathcal{I}$ of type $\tau_1 \times \cdots \times \tau_m$

Guards: $\gamma ::= t_\tau = t_\tau \mid R(t_{\tau_1}, \ldots, t_{\tau_m}) \mid \gamma \wedge \gamma \mid \neg\gamma \mid ite(\gamma : \gamma, \gamma) \mid \exists y : \gamma.\ \gamma$
$\tau \in S \setminus \{\sigma_{\mathsf{S(f)}}\}$, $R \in \mathcal{R}$ of type $\tau_1 \times \cdots \times \tau_m$

Terms: $t_\tau ::= c \mid x \mid f(t_{\tau_1}, \ldots, t_{\tau_m}) \mid ite(\gamma : t_\tau, t_\tau) \mid$
$Sp(\varphi)$ (if $\tau = \sigma_{\mathsf{S(f)}}$) $\mid Sp(t_{\tau'})$ (if $\tau = \sigma_{\mathsf{S(f)}}$)
$\tau, \tau' \in S$ with constants $c$, variables $x$ of type $\tau$,
and functions $f$ of type $\tau_1 \times \cdots \times t_m \rightarrow \tau$

Recursive definitions: $R(\boldsymbol{x}) := \rho_R(\boldsymbol{x})$ with $R \in \mathcal{I}$ of type $\tau_1 \times \cdots \times \tau_m$ with
$\tau_i \in S \setminus \{\sigma_{\mathsf{S(f)}}\}$, FL formula $\rho_R(\boldsymbol{x})$ where all relation symbols
$R' \in \mathcal{I}$ occur only positively or inside a support expression.

**Fig. 1.** Syntax of frame logic: $\gamma$ for guards, $t_\tau$ for terms of sort $\tau$, and general formulas $\varphi$. Guards cannot use inductively defined relations or support expressions.

We consider a universe with a foreground sort and several background sorts, each restricted by individual theories, as described in Section 2. We consider the elements of the foreground sort to be *locations* and consider supports as *sets of locations*, i.e., sets of elements of the foreground sort. We hence introduce a background sort $\sigma_{\mathsf{S(f)}}$; the elements of sort $\sigma_{\mathsf{S(f)}}$ model sets of elements of sort $\sigma_{\mathsf{f}}$. Among the relation symbols in $\mathcal{R}$ there is the relation $\in$ of type $\sigma_{\mathsf{f}} \times \sigma_{\mathsf{S(f)}}$ that is interpreted as the usual element relation. The signature includes the standard operations on sets $\cup$, $\cap$ with the usual meaning, the unary function $\tilde{\cdot}$ that is interpreted as the complement on sets (with respect to the set of foreground elements), and the constant $\emptyset$. For these functions and relations we assume a background theory $B_{\sigma_{\mathsf{S(f)}}}$ that is an axiomatization of the theory of sets. We further assume that the signature does not contain any other function or relation symbols involving the sort $\sigma_{\mathsf{S(f)}}$.

For reasoning about changes of the structure over the locations, we assume that there is a subset $F_{\mathsf{m}} \subseteq F$ of function symbols that are declared mutable. These functions can be used to model mutable pointer fields in the heap that can be manipulated by a program and thus change. Formally, we require that each $f \in F_{\mathsf{m}}$ has at least one argument of sort $\sigma_{\mathsf{f}}$.

For variables, let $Var_\tau$ denote the set of variables of sort $\tau$, where $\tau \in S$. We let $\overline{x}$ abbreviate tuples $x_1, \ldots, x_n$ of variables.

Our frame logic over uninterpreted combinations of theories is a variant of first-order logic with recursive definitions that has an additional operator $Sp(\varphi)$ that assigns to each formula $\varphi$ a set of elements (its support or "heaplet" in the context of heaps) in the foreground universe. So $Sp(\varphi)$ is a term of sort $\sigma_{\mathsf{S(f)}}$.

The intended semantics of $Sp(\varphi)$ (and of the inductive relations) is defined formally as a least fixpoint of a set of equations. This semantics is presented in Section 3.3. In the following, we first define the syntax of the logic, then discuss informally the various design decisions for the semantics of supports, before proceeding to a formal definition of the semantics

#### 3.1 Syntax of Frame Logic (FL)

The syntax of our logic is given in the grammar in Figure 1. This extends FO-RD with the rule for building *support expressions*, which are terms of sort $\sigma_{\mathsf{S(f)}}$ of the form $Sp(\alpha)$ for a formula $\alpha$, or $Sp(t)$ for a term $t$.

The formulas defined by $\gamma$ are used as *guards* in existential quantification and in the if-then-else-operator, which is denoted by *ite*. The restriction compared to general formulas is that guards cannot use inductively defined relations ($R$ ranges only over $\mathcal{R}$ in the rule for $\gamma$, and over $\mathcal{R} \cup \mathcal{I}$ in the rule for $\varphi$), nor terms of sort $\sigma_{\mathsf{S(f)}}$ and thus no support expressions ($\tau$ ranges over $S \setminus \{\sigma_{\mathsf{S(f)}}\}$ in the rules for $\gamma$ and over $S$ in the rule for $\varphi$). The requirement that the guard does not use the inductive relations and support expressions is used later to ensure the existence of least fixpoints for defining semantics of inductive definitions. The semantics of an *ite*-formula $ite(\gamma, \alpha, \beta)$ is the same as the one of $(\gamma \wedge \alpha) \vee (\neg\gamma \wedge \beta)$; however, the *supports* of the two formulas will turn out to be different (i.e., $Sp(ite(\gamma : \alpha, \beta))$ and $Sp((\gamma \wedge \alpha) \vee (\neg\gamma \wedge \beta))$ are different), as explained in Section 3.2. The same is true for existential formulas, i.e., $\exists y : \gamma.\varphi$ has the same semantics as $\exists y.\gamma \wedge \varphi$ but, in general, has a different support.

For recursive definitions (throughout the paper, we use the terms recursive definitions and inductive definitions with the same meaning), we require that the relation $R$ that is defined does not have arguments of sort $\sigma_{\mathsf{S(f)}}$. This is another restriction in order to ensure the existence of a least fixpoint model in the definition of the semantics.[1]

#### 3.2 Semantics of Support Expressions: Design Decisions

We discuss the design decisions that go behind the semantics of the support operator $Sp$ in our logic, and then give an example for the support of an inductive definition. The formal conditions that the supports should satisfy are stated in the equations in Figure 2, and are explained in Section 3.3. Here, we start by an informal discussion.

The first decision is to have every formula uniquely define a support, which roughly captures the subdomain of mutable functions that a formula $\varphi$'s truthhood depends on, and have $Sp(\varphi)$ evaluate to it.

The choice for supports of atomic formulae are relatively clear. An atomic formula of the kind $f(x){=}y$, where $x$ is of the foreground sort and $f$ is a mutable function, has as its support the singleton set containing the location interpreted

[1] It would be sufficient to restrict formulas of the form $R(t_1, \ldots, t_n)$ for inductive relations $R$ to not contain support expressions as subterms.

for $x$. And atomic formulas that do not involve mutable functions over the foreground have an empty support. Supports for terms can also be similarly defined. The support of a conjunction $\alpha \wedge \beta$ should clearly be the union of the supports of the two formulas.

*Remark 1.* In traditional separation logic, each pointer field is stored in a separate location, using integer offsets. However, in our work, we view pointers as references and disallow pointer arithmetic. A more accurate heaplet for such references can be obtained by taking heaplet to be the pair $(x, f)$ (see [30]), capturing the fact that the formula depends only on the field $f$ of $x$. Such accurate heaplets can be captured in FL as well— we can introduce a *non-mutable field lookup pointer* $L_f$ and use $x.L_f.f$ in programs instead of $x.f$.

What should the support of a formula $\alpha \vee \beta$ be? The choice we make here is that its support is the *union* of the supports of $\alpha$ and $\beta$. Note that in a model where $\alpha$ is true and $\beta$ is false, we still include the heaplet of $\beta$ in $Sp(\alpha \vee \beta)$. In a sense, this is an overapproximation of the support as far as frame reasoning goes, as surely preserving the model's definitions on the support of $\alpha$ will preserve the truth of $\alpha$, and hence of $\alpha \vee \beta$.

However, we prefer the support to be the union of the supports of $\alpha$ and $\beta$. We think of the support as the subdomain of the universe that determines the meaning of the formula, whether it be *true* or *false*. Consequently, we would like the support of a formula and its negation to be the same. Given that the support of the negation of a disjunction, being a conjunction, is the union of the frames of $\alpha$ and $\beta$, we would like this to be the support.

Separation logic makes a different design decision. Logical formulas are not associated with tight supports, but rather, the semantics of the formula is defined for models with given supports/heaplets, where the idea of a heaplet is whether it supports the *truthhood* of a formula (and not its falsehood). For example, for a model, the various heaplets that satisfy $\neg(f(x) = y)$ in separation logic would include all heaplets where the location of $x$ is not present, which does not coincide with the notion we have chosen for supports. However, for positive formulas, separation logic handles supports more accurately, as it can associate several supports for a formula, yielding two heaplets for formulas of the form $\alpha \vee \beta$ when they are both true in a model. The decision to have a single support for a formula compels us to take the union of the supports to be the support of a disjunction.

There are situations, however, where there are disjunctions $\alpha \vee \beta$, where only *one* of the disjuncts can possibly be true, and hence we would like the support of the formula to be the support of the disjunct that happens to be true. We therefore introduce a new syntactical form $ite(\gamma : \alpha, \beta)$ in frame logic, whose heaplet is the union of the supports of $\gamma$ and $\alpha$, if $\gamma$ is true, and the supports of $\gamma$ and $\beta$ if $\gamma$ is false. While the truthhood of $ite(\gamma : \alpha, \beta)$ is the same as that of $(\gamma \wedge \alpha) \vee (\neg\gamma \wedge \beta)$, its supports are potentially smaller, allowing us to write formulas with tighter supports to support better frame reasoning. Note that the support of $ite(\gamma : \alpha, \beta)$ and its negation $ite(\gamma : \neg\alpha, \neg\beta)$ are the same, as we desired.

Turning to quantification, the support for a formula of the form $\exists x.\alpha$ is hard to define, as its truthhood could depend on the entire universe. We hence provide a mechanism for *guarded* quantification, in the form $\exists x : \gamma.\ \ \alpha$. The semantics of this formula is that there exists some location that satisfies the guard $\gamma$, for which $\alpha$ holds. The support for such a formula includes the support of the guard, and the supports of $\alpha$ when $x$ is interpreted to be a location that satisfies $\gamma$. For example, $\exists x : (x = f(y)).\ \ g(x) = z$ has as its support the locations interpreted for $y$ and $f(y)$ only.

For a formula $R(\overline{t})$ with an inductive relation $R$ defined by $R(\overline{x}) := \rho_R(\overline{x})$, the support descends into the definition, changing the variable assignment of the variables in $\overline{x}$ from the inductive definition to the terms in $\overline{t}$. Furthermore, it contains the elements to which mutable functions are applied in the terms in $\overline{t}$.

Recursive definitions are designed such that the evaluation of the equations for the support expressions is independent of the interpretation of the inductive relations. The equations mainly depend on the syntactic structure of formulas and terms. Only the semantics of guards, and the semantics of subterms under a mutable function symbol play a role. For this reason, we disallow guards to contain recursively defined relations or support expressions. We also require that the only functions involving the sort $\sigma_{\mathsf{S(f)}}$ are the standard functions involving sets. Thus, subterms of mutable functions cannot contain support expressions (which are of sort $\sigma_{\mathsf{S(f)}}$) as subterms.

These restrictions ensure that there indeed exists a unique simultaneous least solution of the equations for the inductive relations and the support expressions.

We end this section with an example.

*Example 1.* Consider the definition of a predicate $\mathit{tree}(x)$ w.r.t. two unary mutable functions *left* and *right*:

$$\begin{aligned} \mathit{tree}(x) &:= \mathit{ite}(x = \mathit{nil} : \mathit{true}, \alpha) \text{ where} \\ \alpha &= \exists \ell, r : (\ell = \mathit{left}(x) \wedge r = \mathit{right}(x)).\mathit{tree}(\ell) \wedge \mathit{tree}(r) \wedge \\ &\quad \mathit{Sp}(\mathit{tree}(\ell)) \cap \mathit{Sp}(\mathit{tree}(r)) = \emptyset \wedge \neg(x \in \mathit{Sp}(\mathit{tree}(\ell)) \cup \mathit{Sp}(\mathit{tree}(r))) \end{aligned}$$

This inductive definition defines binary trees with pointer fields *left* and *right* for left- and right-pointers, by stating that $x$ points to a tree if either $x$ is equal to *nil* (in this case its support is empty), or $\mathit{left}(x)$ and $\mathit{right}(x)$ are trees with disjoint supports. The last conjunct says that $x$ does not belong to the support of the left and right subtrees; this condition is, strictly speaking, not required to define trees (under least fixpoint semantics). Note that the access to the support of formulas eases defining disjointness of heaplets, like in separation logic. The support of $\mathit{tree}(x)$ turns out to be precisely the nodes that are reachable from $x$ using *left* and *right* pointers, as one would desire. Consequently, if a pointer outside this support changes, we would be able to conclude using frame reasoning that the truth value of $\mathit{tree}(x)$ does not change. □

#### 3.3 Formal Semantics of Frame Logic

Before we explain the semantics of the support expressions and inductive definitions, we introduce a semantics that treats support expressions and the symbols

$$
\begin{aligned}
[\![Sp(c)]\!]_M(\nu) &= [\![Sp(x)]\!]_M(\nu) = \emptyset \text{ for a constant } c \text{ or variable } x\\
[\![Sp(f(t_1,\ldots,t_n))]\!]_M(\nu) &= \begin{cases} \bigcup\limits_{i \text{ with } t_i \text{ of sort } \sigma_{\mathsf{f}}} \{[\![t_i]\!]_{M,\nu}\} \cup \bigcup\limits_{i=1}^{n} [\![Sp(t_i)]\!]_M(\nu) & \text{if } f \in F_{\mathsf{m}}\\ \bigcup\limits_{i=1}^{n} [\![Sp(t_i)]\!]_M(\nu) & \text{if } f \notin F_{\mathsf{m}} \end{cases}\\
[\![Sp(Sp(\varphi))]\!]_M(\nu) &= [\![Sp(\varphi)]\!]_M(\nu)\\
[\![Sp(Sp(t))]\!]_M(\nu) &= [\![Sp(t)]\!]_M(\nu)\\
[\![Sp(t_1 = t_2)]\!]_M(\nu) &= [\![Sp(t_1)]\!]_M(\nu) \cup [\![Sp(t_2)]\!]_M(\nu)\\
[\![Sp(R(t_1,\ldots,t_n))]\!]_M(\nu) &= \textstyle\bigcup_{i=1}^{n} [\![Sp(t_i)]\!]_M(\nu) \text{ for } R \in \mathcal{R}\\
[\![Sp(R(\overline{t}))]\!]_M(\nu) &= [\![Sp(\rho_R(\overline{x}))]\!]_M(\nu[\overline{x} \leftarrow [\![\overline{t}]\!]_{M,\nu}]) \cup \textstyle\bigcup_{i=1}^{n} [\![Sp(t_i)]\!]_M(\nu)\\
&\quad \text{for } R \in \mathcal{I} \text{ with definition } R(\overline{x}) := \rho_R(\overline{x}),\\
&\quad \overline{t} = (t_1,\ldots,t_n), \overline{x} = (x_1,\ldots,x_n)\\
[\![Sp(\alpha \wedge \beta)]\!]_M(\nu) &= [\![Sp(\alpha)]\!]_M(\nu) \cup [\![Sp(\beta)]\!]_M(\nu)\\
[\![Sp(\neg\varphi)]\!]_M(\nu) &= [\![Sp(\varphi)]\!]_M(\nu)\\
[\![Sp(ite(\gamma : \alpha, \beta))]\!]_M(\nu) &= [\![Sp(\gamma)]\!]_M(\nu) \cup \begin{cases} [\![Sp(\alpha)]\!]_M(\nu) \text{ if } M, \nu \models \gamma\\ [\![Sp(\beta)]\!]_M(\nu) \text{ if } M, \nu \not\models \gamma \end{cases}\\
[\![Sp(ite(\gamma : t_1, t_2))]\!]_M(\nu) &= [\![Sp(\gamma)]\!]_M(\nu) \cup \begin{cases} [\![Sp(t_1)]\!]_M(\nu) \text{ if } M, \nu \models \gamma\\ [\![Sp(t_2)]\!]_M(\nu) \text{ if } M, \nu \not\models \gamma \end{cases}\\
[\![Sp(\exists y : \gamma.\varphi)]\!]_M(\nu) &= \bigcup_{u \in D_y} [\![Sp(\gamma)]\!]_M(\nu[y \leftarrow u]) \cup \bigcup_{u \in D_y; M, \nu[y \leftarrow u] \models \gamma} [\![Sp(\varphi)]\!]_M(\nu[y \leftarrow u])
\end{aligned}
$$

**Fig. 2.** Equations for support expressions from $\mathcal{I}$ as uninterpreted symbols. We refer to this semantics as *uninterpreted semantics*. For the formal definition we need to introduce some terminology first.

An occurrence of a variable $x$ in a formula is free if it does not occur under the scope of a quantifier for $x$. By renaming variables we can assume that each variable only occurs freely in a formula or is quantified by exactly one quantifier in the formula. We write $\varphi(x_1,\ldots,x_k)$ to indicate that the free variables of $\varphi$ are among $x_1,\ldots,x_k$. Substitution of a term $t$ for all free occurrences of variable $x$ in a formula $\varphi$ is denoted $\varphi[t/x]$. Multiple variables are substituted simultaneously as $\varphi[t_1/x_1,\ldots,t_n/x_n]$. We abbreviate this by $\varphi[\overline{t}/\overline{x}]$.

A model is of the form $M = (U; [\![\cdot]\!]_M)$ where $U = (U_\sigma)_{\sigma \in S}$ contains a universe for each sort, and an interpretation function $[\![\cdot]\!]_M$. The universe for the sort $\sigma_{\mathsf{S(f)}}$ is the powerset of the universe for $\sigma_{\mathsf{f}}$.

A variable assignment is a function $\nu$ that assigns to each variable a concrete element from the universe for the sort of the variable. For a variable $x$, we write $D_x$ for the universe of the sort of $x$ (the domain of $x$). For a variable $x$ and an element $u \in D_x$ we write $\nu[x \leftarrow u]$ for the variable assignment that is obtained from $\nu$ by changing the value assigned for $x$ to $u$.

The interpretation function $[\![\cdot]\!]_M$ maps each constant $c$ of sort $\sigma$ to an element $[\![c]\!]_M \in U_\sigma$, each function symbol $f : \tau_1 \times \ldots \times \tau_m \to \tau$ to a concrete function $[\![f]\!]_M : U_{\tau_1} \times \ldots \times U_{\tau_m} \to U_\tau$, and each relation symbol $R \in \mathcal{R} \cup \mathcal{I}$ of type $\tau_1 \times \ldots \times \tau_m$ to a concrete relation $[\![R]\!]_M \subseteq U_{\tau_1} \times \ldots \times U_{\tau_m}$. These interpretations are assumed to satisfy the background theories (see Section 2). Furthermore, the interpretation function maps each expression of the form $\mathit{Sp}(\varphi)$ to a function $[\![\mathit{Sp}(\varphi)]\!]_M$ that assigns to each variable assignment $\nu$ a set $[\![\mathit{Sp}(\varphi)]\!]_M(\nu)$ of foreground elements. The set $[\![\mathit{Sp}(\varphi)]\!]_M(\nu)$ corresponds to the support of the formula when the free variables are interpreted by $\nu$. Similarly, $[\![\mathit{Sp}(t)]\!]_M$ is a function from variable assignments to sets of foreground elements.

Based on such models, we can define the semantics of terms and formulas in the standard way. The only construct that is non-standard in our logic are terms of the form $\mathit{Sp}(\varphi)$, for which the semantics is directly given by the interpretation function. We write $[\![t]\!]_{M,\nu}$ for the interpretation of a term $t$ in $M$ with variable assignment $\nu$. With this convention, $[\![\mathit{Sp}(\varphi)]\!]_M(\nu)$ denotes the same thing as $[\![\mathit{Sp}(\varphi)]\!]_{M,\nu}$. As usual, we write $M, \nu \models \varphi$ to indicate that the formula $\varphi$ is true in $M$ with the free variables interpreted by $\nu$, and $[\![\varphi]\!]_M$ denotes the relation defined by the formula $\varphi$ with free variables $\overline{x}$.

We refer to the above semantics as the *uninterpreted semantics* of $\varphi$ because we do not give a specific meaning to inductive definitions and support expressions.

Now let us define the true semantics for FL. The relation symbols $R \in \mathcal{I}$ represent inductively defined relations, which are defined by equations of the form $R(\overline{x}) := \rho_R(\overline{x})$ (see Figure 1). In the intended meaning, $R$ is interpreted as the least relation that satisfies the equation

$$[\![R(\overline{x})]\!]_M = [\![\rho_R(\overline{x})]\!]_M.$$

The usual requirement for the existence of a unique least fixpoint of the equation is that the definition of $R$ does not negatively depend on $R$. For this reason, we require that in $\rho_R(\overline{x})$ each occurrence of an inductive predicate $R' \in \mathcal{I}$ is either inside a support expression, or it occurs under an even number of negations.[2]

Every support expression is evaluated on a model to a set of foreground elements (under a given variable assignment $\nu$). Formally, we are interested in models in which the support expressions are interpreted to be the sets that correspond to the *smallest solution of the equations given in Figure 2*. The intuition behind these definitions was explained in Section 3.2

*Example 2.* Consider the inductive definition $\mathit{tree}(x)$ defined in Example 1. To check whether the equations from Figure 2 indeed yield the desired support, note that the supports of $\mathit{Sp}(x = \mathit{nil}) = \mathit{Sp}(x) = \mathit{Sp}(\mathit{true}) = \emptyset$. Below, we write $[u]$ for a variable assignment that assigns $u$ to the free variable of the formula that we are considering. Then we obtain that $\mathit{Sp}(\mathit{tree}(x))[u] = \emptyset$ if $u = \mathit{nil}$, and $\mathit{Sp}(\mathit{tree}(x))[u] = \mathit{Sp}(\alpha)[u]$ if $x \neq \mathit{nil}$. The formula $\alpha$ is existentially quantified with guard $\ell = \mathit{left}(x) \wedge r = \mathit{right}(x)$. The support of this guard is $\{u\}$ because mutable functions are applied to $x$. The support of the remaining part of $\alpha$ is the union of the supports of $\mathit{tree}(\ell)[\mathit{left}(u)]$ and $\mathit{tree}(r)[\mathit{right}(u)]$ (the assignments for $\ell$ and $r$ that make the guard true). So we obtain for the case that $u \neq \mathit{nil}$ that the element $u$ enters the support, and the recursion further descends into the subtrees of $u$, as desired. □

[2] As usual, it would be sufficient to forbid negative occurrences of inductive predicates in mutual recursion.

A *frame model* is a model in which the interpretation of the inductive relations and of the support expressions corresponds to the least solution of the respective equations (see the Technical Report [25] for a rigorous formalisation).

**Proposition 1.** *For each model $M$, there is a unique frame model over the same universe and the same interpretation of the constants, functions, and non-inductive relations.*

#### 3.4 A Frame Theorem

The support of a formula can be used for frame reasoning in the following sense: if we modify a model $M$ by changing the interpretation of the mutable functions (e.g., a program modifying pointers), then truth values of formulas do not change if the change happens outside the support of the formula. This is formalized below and proven in the Technical Report [25].

Given two models $M, M'$ over the same universe, we say that $M'$ is a *mutation of* $M$ if $[\![R]\!]_M = [\![R]\!]_{M'}$, $[\![c]\!]_M = [\![c]\!]_{M'}$, and $[\![f]\!]_M = [\![f]\!]_{M'}$, for all constants $c$, relations $R \in \mathcal{R}$, and functions $f \in F \setminus F_{\mathsf{m}}$. In other words, $M$ can only be different from $M'$ on the interpretations of the mutable functions, the inductive relations, and the support expressions.

Given a subset $X \subseteq U_{\sigma_{\mathsf{f}}}$ of the elements from the foreground universe, we say that the *mutation is stable on* $X$ if the values of the mutable functions did not change on arguments from $X$, that is, $[\![f]\!]_M(u_1, \ldots, u_n) = [\![f]\!]_{M'}(u_1, \ldots, u_n)$ for all mutable functions $f \in F_{\mathsf{m}}$ and all appropriate tuples $u_1, \ldots, u_n$ of arguments with $\{u_1, \ldots, u_n\} \cap X \neq \emptyset$.

**Theorem 1 (Frame Theorem).** *Let $M, M'$ be frame models such that $M'$ is a mutation of $M$ that is stable on $X \subseteq U_{\sigma_f}$, and let $\nu$ be a variable assignment. Then $M, \nu \models \alpha$ iff $M', \nu \models \alpha$ for all formulas $\alpha$ with $[\![Sp(\alpha)]\!]_M(\nu) \subseteq X$, and $[\![t]\!]_{M,\nu} = [\![t]\!]_{M',\nu}$ for all terms $t$ with $[\![Sp(t)]\!]_M(\nu) \subseteq X$.*

#### 3.5 Reduction from Frame Logic to FO-RD

The only extension of frame logic compared to FO-RD is the operator $Sp$, which defines a function from interpretations of free variables to sets of foreground elements. The semantics of this operator can be captured within FO-RD itself, so reasoning within frame logic can be reduced to reasoning within FO-RD.

A formula $\alpha(\overline{y})$ with $\overline{y} = y_1, \ldots, y_m$ has one support for each interpretation of the free variables. We capture these supports by an inductively defined relation $Sp_\alpha(\overline{y}, z)$ of arity $m+1$ such that for each frame model $M$, we have $(u_1, \ldots, u_m, u) \in [\![Sp_\alpha]\!]_M$ if $u \in [\![Sp(\alpha)]\!]_M(\nu)$ for the interpretation $\nu$ that interprets $y_i$ as $u_i$.

Since the semantics of $Sp(\alpha)$ is defined over the structure of $\alpha$, we introduce corresponding inductively defined relations $Sp_\beta$ and $Sp_t$ for all subformulas $\beta$ and subterms $t$ of either $\alpha$ or of a formula $\rho_R$ for $R \in \mathcal{I}$.

$$
\begin{aligned}
\mathit{list}(x) &:= \mathit{ite}(x = \mathit{nil}, \mathit{true}, \exists z : z = \mathit{next}(x).\ \mathit{list}(z) \wedge x \notin \mathit{Sp}(\mathit{list}(z)) \\
&\hfill \text{(linked list)} \\
\mathit{dll}(x) &:= \mathit{ite}(x = \mathit{nil} : \top, \mathit{ite}(\mathit{next}(x) = \mathit{nil} : \top, \exists z : z = \mathit{next}(x). \\
&\quad \mathit{prev}(z) = x \wedge \mathit{dll}(z) \wedge x \notin \mathit{Sp}(\mathit{dll}(z)))) \qquad \text{(doubly linked list)} \\
\mathit{lseg}(x, y) &:= \mathit{ite}(x = y : \top, \exists z : z = \mathit{next}(x).\ \mathit{lseg}(z, y) \wedge x \notin \mathit{Sp}(\mathit{lseg}(z, y))) \\
&\hfill \text{(linked list segment)} \\
\mathit{length}(x, n) &:= \mathit{ite}(x = \mathit{nil} : n = 0, \exists z : z = \mathit{next}(x).\ \mathit{length}(z, n - 1)) \\
&\hfill \text{(length of list)} \\
\mathit{slist}(x) &:= \mathit{ite}(x = \mathit{nil} : \top, \mathit{ite}(\mathit{next}(x) = \mathit{nil}, \top, \exists z : z = \mathit{next}(x). \\
&\quad \mathit{key}(x) \leq \mathit{key}(z) \wedge \mathit{slist}(z) \wedge x \notin \mathit{Sp}(\mathit{slist}(z)))) \qquad \text{(sorted list)} \\
\mathit{mkeys}(x, M) &:= \mathit{ite}(x = \mathit{nil} : M = \emptyset, \exists z, M_1 : z = \mathit{next}(x). \\
&\quad M = M_1 \cup_m \{\mathit{key}(x)\} \wedge \mathit{mkeys}(z, M_1)) \wedge x \notin \mathit{Sp}(\mathit{mkeys}(z, M_1)) \\
&\hfill \text{(multiset of keys in linked list)} \\
\mathit{btree}(x) &:= \mathit{ite}(x = \mathit{nil} : \top, \exists \ell, r : \ell = \mathit{left}(x) \wedge r = \mathit{right}(x). \\
&\quad \mathit{btree}(\ell) \wedge \mathit{btree}(r) \wedge x \notin \mathit{Sp}(\mathit{btree}(\ell)) \wedge x \notin \mathit{Sp}(\mathit{btree}(r)) \wedge \\
&\quad \mathit{Sp}(\mathit{btree}(\ell)) \cap \mathit{Sp}(\mathit{btree}(r)) = \emptyset) \qquad \text{(binary tree)} \\
\mathit{bst}(x) &:= \mathit{ite}(x = \mathit{nil} : \top, \mathit{ite}(\mathit{left}(x) = \mathit{nil} \wedge \mathit{right}(x) = \mathit{nil} : \top, \mathit{ite}(\mathit{left}(x) = \mathit{nil} : \\
&\quad \exists r : r = \mathit{right}(x).\ \mathit{key}(x) \leq \mathit{key}(r) \wedge \mathit{bst}(r) \wedge x \notin \mathit{Sp}(\mathit{bst}(r)), \\
&\quad \mathit{ite}(\mathit{right}(x) = \mathit{nil} : \exists \ell : \ell = \mathit{left}(x).\ \mathit{key}(\ell) \leq \mathit{key}(x) \wedge \mathit{bst}(\ell) \wedge x \notin \mathit{Sp}(\mathit{bst}(\ell)), \\
&\quad \exists \ell, r : \ell = \mathit{left}(x) \wedge r = \mathit{right}(x).\ \mathit{key}(x) \leq \mathit{key}(r) \wedge \mathit{key}(\ell) \leq \mathit{key}(x) \wedge \\
&\quad \mathit{bst}(\ell) \wedge \mathit{bst}(r) \ \wedge x \notin \mathit{Sp}(\mathit{bst}(\ell)) \wedge x \notin \mathit{Sp}(\mathit{bst}(r)) \wedge \\
&\quad \mathit{Sp}(\mathit{bst}(\ell)) \cap \mathit{Sp}(\mathit{bst}(r)) = \emptyset)))) \qquad \text{(binary search tree)} \\
\mathit{height}(x, n) &:= \mathit{ite}(x = \mathit{nil} : n = 0, \exists \ell, r, n_1, n_2 : \ell = \mathit{left}(x) \wedge r = \mathit{right}(x). \\
&\quad \mathit{height}(\ell, n_1) \wedge \mathit{height}(r, n_2) \wedge \mathit{ite}(n_1 > n_2 : n = n_1 + 1, n = n_2 + 1)) \\
&\hfill \text{(height of binary tree)} \\
\mathit{bfac}(x, b) &:= \mathit{ite}(x = \mathit{nil} : 0, \exists \ell, r, n_1, n_2 : \ell = \mathit{left}(x) \wedge r = \mathit{right}(x). \\
&\quad \mathit{height}(\ell, n_1) \wedge \mathit{height}(r, n_2) \wedge b = n_2 - n_1) \\
&\hfill \text{(balance factor (for AVL tree))} \\
\mathit{avl}(x) &:= \mathit{ite}(x = \mathit{nil} : \top, \exists \ell, r : \ell = \mathit{left}(x) \wedge r = \mathit{right}(x). \\
&\quad \mathit{avl}(\ell) \wedge \mathit{avl}(r) \wedge \mathit{bfac}(x) \in \{-1, 0, 1\} \wedge \\
&\quad x \notin \mathit{Sp}(\mathit{avl}(\ell)) \cup \mathit{Sp}(\mathit{avl}(r)) \wedge \mathit{Sp}(\mathit{avl}(\ell)) \cap \mathit{Sp}(\mathit{avl}(r)) = \emptyset) \quad \text{(avl tree)} \\
\mathit{ttree}(x) &:= \mathit{pttree}(x, \mathit{nil}) \qquad \text{(threaded tree)} \\
\mathit{pttree}(x, p) &:= \mathit{ite}(x = \mathit{nil} : \top, \exists \ell, r : \ell = \mathit{left}(x) \wedge r = \mathit{right}(x). \\
&\quad ((r = \mathit{nil} \wedge \mathit{tnext}(x) = p) \vee (r \neq \mathit{nil} \wedge \mathit{tnext}(x) = r)) \wedge \\
&\quad \mathit{pttree}(\ell, x) \wedge \mathit{pttree}(r, p) \wedge x \notin \mathit{Sp}(\mathit{pttree}(\ell, x)) \cup \mathit{Sp}(\mathit{pttree}(r, p)) \wedge \\
&\quad \mathit{Sp}(\mathit{pttree}(\ell, x)) \cap \mathit{Sp}(\mathit{pttree}(r, p)) = \emptyset) \\
&\hfill \text{(threaded tree auxiliary definition)}
\end{aligned}
$$

**Fig. 3.** Example definitions of data-structures and other predicates in Frame Logic

The equations for supports from Figure 2 can be expressed by inductive definitions for the relations $Sp_\beta$. The translations are shown in the Technical Report [25]. It is not hard to see that general frame logic formulas can be translated to FO-RD formulas that make use of these new inductively defined relations.

**Proposition 2.** *For every frame logic formula there is an equisatisfiable FO-RD formula with the signature extended by auxiliary predicates for recursive definitions of supports.*

#### 3.6 Expressing Data-Structures Properties in FL

We now present the formulation of several data-structures and properties about them in FL. Figure 3 depicts formulations of singly- and doubly-linked lists, list segments, lengths of lists, sorted lists, the multiset of keys stored in a list (assuming a background sort of multisets), binary trees, their heights, and AVL trees. In all these definitions, the support operator plays a crucial role. We also present a formulation of *single threaded binary trees* (adapted from [7]), which are binary trees where, apart from tree-edges, there is a pointer *tnext* that connects every tree node to the inorder successor in the tree; these pointers go from leaves to ancestors arbitrarily far away in the tree, making it a nontrivial definition.

We believe that FL formulas naturally and succinctly express these data-structures and their properties, making it an attractive logic for annotating programs.

### 4 Programs and Proofs

In this section, we develop a program logic for a while-programming language that can destructively update heaps. We assume that location variables are denoted by variables of the form $x$ and $y$, whereas variables that denote other data (which would correspond to the *background* sorts in our logic) are denoted by $v$. We omit the grammar to construct background terms and formulas, and simply denote such 'background expressions' with *be* and clarify the sort when it is needed. Finally, we assume that our programs are written in Single Static Assignment (SSA) form, which means that every variable is assigned to at most once in the program text. The grammar for our programming language is in Figure 4.

$$
\begin{aligned}
S ::= {} & x := c \mid x := y \mid x := y.f \mid v := be \mid x.f := y \\
& \mid \mathsf{alloc}(x) \mid \mathsf{free}(x) \mid \mathsf{if}\ be\ \mathsf{then}\ S\ \mathsf{else}\ S \mid \mathsf{while}\ be\ \mathsf{do}\ S \mid S\,;\,S
\end{aligned}
$$

**Fig. 4.** Grammar of while programs. $c$ is a constant location, $f$ is a field pointer, and *be* is a background expression. In our logic, we model every field $f$ as a function $f()$ from locations to the appropriate sort.

#### 4.1 Operational Semantics

A configuration $\mathcal{C}$ is of the form $(M, H, U)$ where $M$ contains interpretations for the store and the heap. The store is a partial map that interprets variables, constants, and non-mutable functions (a function from location variables to locations) and the heap is a total map on the domain of locations that interprets mutable functions (a function from pointers and locations to locations). $H$ is a subset of locations denoting the set of allocated locations, and $U$ is a subset of locations denoting *a subset* of unallocated locations that can be allocated in the future. We introduce a special configuration $\bot$ that the program transitions to when it dereferences a variable not in $H$.

A configuration $(M, H, U)$ is *valid* if all variables of the location sort map only to locations not in $U$, locations in $H$ do not point to any location in $U$, and $U$ is a subset of the complement of $H$ that does not contain *nil* or the locations mapped to by the variables. We denote this by $\mathit{valid}(M, H, U)$. Initial configurations and reachable configurations of any program will be valid.

The transition of configurations on various commands that manipulate the store and heap are defined in the natural way. Allocation adds a new location from $U$ into $H$ with pointer-fields defaulting to *nil* and default data fields. See the Technical Report [25] for more details.

#### 4.2 Triples and Validity

We express specifications of programs using triples of the form $\{\alpha\}S\{\beta\}$ where $\alpha$ and $\beta$ are FL formulae and $S$ is a program. The formulae are, however, restricted— for simplicity, we disallow atomic relations on locations, and functions with arity greater than one. We also disallow functions from a background sort to the foreground sort (see Section 3). Lastly, quantified formulae can have supports as large as the entire heap. However, our program logic covers a more practical fragment without compromising expressivity. Thus, we require guards in quantification to be of the form $f(z') = z$ or $z \in U$ ($z$ is the quantified variable).

We define a triple to be *valid* if every valid configuration with heaplet being precisely the support of $\alpha$, when acted on by the program, yields a configuration with heaplet being the support of $\beta$. More formally, a triple is valid if for every valid configuration $(M, H, U)$ such that $M \models \alpha$, $H = [\![Sp(\alpha)]\!]_M$:

- it is never the case that the abort state $\bot$ is encountered in the execution on $S$.
- if $(M, H, U)$ transitions to $(M', H', U')$ on $S$, then $M' \models \beta$ and $H' = [\![Sp(\beta)]\!]_{M'}$

#### 4.3 Program Logic

First, we define a set of *local rules* and rules for conditionals, while, sequence, consequence, and framing:

**Assignment:** $\{true\}\ x := y\ \{x = y\} \qquad \{true\}\ x := c\ \{x = c\}$

**Lookup:** $\{f(y) = f(y)\}\ x := y.f\ \{x = f(y)\}$

**Mutation:** $\{f(x) = f(x)\}\ x.f := y\ \{f(x) = y\}$

**Allocation:** $\{true\}\ \mathsf{alloc}(x)\ \{\bigwedge_{f \in F} f(x) = def_f\}$

**Deallocation:** $\{f(x) = f(x)\}\ \mathsf{free}(x)\ \{true\}$

**Conditional:**
$$\frac{\{be \wedge \alpha\}\ S\ \{\beta\} \quad \{\neg be \wedge \alpha\}\ T\ \{\beta\}}{\{\alpha\}\ \mathsf{if}\ be\ \mathsf{then}\ S\ \mathsf{else}\ T\ \{\beta\}}$$

**While:**
$$\frac{\{\alpha \wedge be\}\ S\ \{\alpha\}}{\{\alpha\}\ \mathsf{while}\ be\ \mathsf{do}\ S\ \{\neg be \wedge \alpha\}}$$

**Sequence:**
$$\frac{\{\alpha\}\ S\ \{\beta\} \quad \{\beta\}\ T\ \{\mu\}}{\{\alpha\}\ S\ ;\ T\ \{\mu\}}$$

**Consequence:**
$$\frac{\begin{array}{c} \alpha' \implies \alpha \\ \beta \implies \beta' \end{array} \quad \{\alpha\}\ S\ \{\beta\} \quad \begin{array}{c} Sp(\alpha) = Sp(\alpha') \\ Sp(\beta) = Sp(\beta') \end{array}}{\{\alpha'\}\ S\ \{\beta'\}}$$

**Frame:**
$$\frac{Sp(\alpha) \cap Sp(\mu) = \emptyset \quad \{\alpha\}\ S\ \{\beta\}}{\{\alpha \wedge \mu\}\ S\ \{\beta \wedge \mu\}}\ vars(S) \cap fv(\mu) = \emptyset$$

The above rules are intuitively clear and are similar to the local rules in separation logic [38]. The rules for statements capture their semantics using minimal/tight heaplets, and the frame rule allows proving triples with larger heaplets. In the rule for alloc, the postcondition says that the newly allocated location has default values for all pointer fields and datafields (denoted as $def_f$). The soundness of the frame rule relies crucially on the frame theorem for FL (Theorem 1). The full soundness proof can be found in the Technical Report [25].

**Theorem 2.** *The above rules are sound with respect to the operational semantics.*

#### 4.4 Weakest-Precondition Proof Rules

We now turn to the much more complex problem of designing rules that give weakest preconditions for arbitrary postconditions, for loop-free programs. In separation logic, such rules resort to using the magic-wand operator $-\!*$ [12, 27, 28, 38], The magic-wand operator, a complex operator whose semantics calls for *second-order quantification* over arbitrarily large submodels. In our setting, our main goal is to show that FL is itself capable of expressing weakest preconditions of postconditions written in FL.

First, we define a notion of *Weakest Tightest Precondition* (WTP) of a formula $\beta$ with respect to each command in our operational semantics. To define this notion, we first define a preconfiguration, and use that definition to define weakest tightest preconditions:

**Definition 1.** *The preconfigurations corresponding to a valid configuration $(M, H, U)$ with respect to a program $S$ are a set of valid configurations of the form $(M_p, H_p, U_p)$ (with $M_p$ being a model, $H_p$ and $U_p$ a subuniverse of the locations in $M_p$, and $U_p$ being unallocated locations) such that when $S$ is executed on $M_p$ with unallocated set $U_p$ it dereferences only locations in $H_p$ and results (using the operational semantics rules) in $(M, H, U)$ or gets stuck (no transition is available). That is:*

$$\begin{aligned} &\mathit{preconfigurations}((M,H,U),S) = \\ &\{(M_p,H_p,U_p) \mid \mathit{valid}(M_p,H_p,U_p) \text{ and } (M_p,H_p,U_p) \overset{S}{\Rightarrow} (M,H,U) \text{ or} \\ &\qquad (M_p,H_p,U_p) \text{ gets stuck on } S\} \end{aligned}$$

**Definition 2.** *$\alpha$ is a WTP of a formula $\beta$ with respect to a program $S$ if*

$$\begin{aligned} &\{(M_p,H_p,U_p) \mid M_p \models \alpha, H_p = [\![Sp(\alpha)]\!]_{M_p}, \mathit{valid}(M_p,H_p,U_p)\} \\ &= \{\mathit{preconfigurations}((M,H,U),S) \mid M \models \beta, H = [\![Sp(\beta)]\!]_M, \mathit{valid}(M,H,U)\} \end{aligned}$$

With the notion of weakest tightest preconditions, we define global program logic rules for each command of our language. In contrast to local rules, global specifications contain heaplets that may be larger than the smallest heap on which one can execute the command.

Intuitively, a WTP of $\beta$ for lookup states that $\beta$ must hold in the precondition when $x$ is interpreted as $x'$, where $x' = f(y)$, and further that the location $y$ must belong to the support of $\beta$. The rules for mutation and allocation are more complex. For mutation, we define a transformation $MW^{x.f:=y}(\beta)$ that evaluates a formula $\beta$ in the pre-state as though it were evaluated in the post-state. We similarly define such a transformation $MW_v^{\mathsf{alloc}(x)}$ for allocation. We will define these in detail later. Finally, the deallocation rule ensures $x$ is not in the support of the postcondition. The conjunct $f(x) = f(x)$ is provided to satisfy the tightness condition, ensuring the support of the precondition is the support of the postcondition with $x$ added. The rules can be seen below, and the proof of soundness for these global rules can be found in the Technical Report [25].

**Assignment-G:** $\{\beta[y/x]\}\ x := y\ \{\beta\} \qquad \{\beta[c/x]\}\ x := c\ \{\beta\}$

**Lookup-G:** $\{\exists x' : x' = f(y).\ (\beta \wedge y \in Sp(\beta))[x'/x]\}\ x := y.f\ \{\beta\}$
(where $x'$ does not occur in $\beta$)

**Mutation-G:** $\{MW^{x.f:=y}(\beta \wedge x \in Sp(\beta))\}\ x.f := y\ \{\beta\}$

**Allocation-G:** $\{\forall v : (v \in U).(v \neq nil \Rightarrow MW_v^{\mathsf{alloc}(x)}(\beta))\}\ \mathsf{alloc}(x)\ \{\beta\}$
(for some fresh variable $v$)

**Deallocation-G:** $\{\beta \wedge x \notin Sp(\beta) \wedge f(x) = f(x)\}\ \mathsf{free}(x)\ \{\beta\}$
(where $f \in F_{\mathsf{m}}$ is an arbitrary (unary) mutable function)

#### 4.5 Definitions of *MW* Primitives

Recall that the $MW$[3] primitives $MW^{x.f:=y}$ and $MW_v^{\mathsf{alloc}(x)}$ need to evaluate a formula $\beta$ in the pre-state as it would evaluate in the post-state after mutation and allocation statements. The definition of $MW^{x.f:=y}$ is as follows:

$$MW^{x.f:=y}(\beta) = \beta[\lambda z.\ ite(z = x : ite(f(x) = f(x) : y, y), f(z))/f]$$

The $\beta[\lambda z.\rho(z)/f]$ notation is shorthand for saying that each occurrence of a term of the form $f(t)$, where $t$ is a term, is substituted (recursively, from inside out) by the term $\rho(t)$. The precondition essentially evaluates $\beta$ taking into account $f$'s transformation, but we use the *ite* expression with a tautological guard $f(x) = f(x)$ (which has the support containing the singleton $x$) in order to preserve the support. The definition of $MW_v^{\mathsf{alloc}(x)}$ is similar. Refer to the Technical Report [25] for details.

**Theorem 3.** *The rules above suffixed with **-G** are sound w.r.t the operational semantics. And, each precondition corresponds to the weakest tightest precondition of $\beta$.*

#### 4.6 Example

In this section, we will see an example of using our program logic rules that we described earlier. This will demonstrate the utility of Frame Logic as a logic for annotating and reasoning with heap manipulating programs, as well as offer some intuition about how our program logic can be deployed in a practical setting. The following program performs in-place list reversal: `j := nil ; while (i != nil) do k := i.next ; i.next := j ; j := i ; i := k` For the sake of simplicity, instead of proving that this program reverses a list, we will instead prove the simpler claim that after executing this program $j$ is a *list*. The recursive definition of *list* we use for this proof is the one from Figure 3:

$$list(x) := ite(x = nil, true, \exists z : z = next(x).\ list(z) \wedge x \notin Sp(list(z)))$$

We need to also give an invariant for the while loop, simply stating that $i$ and $j$ point to disjoint lists: $list(i) \wedge list(j) \wedge Sp(list(i)) \cap Sp(list(j)) = \emptyset$.

We prove that this is indeed an invariant of the while loop below. Our proof uses a mix of both local and global rules from Sections 4.3 and 4.4 above to demonstrate how either type of rule can be used. We also use the consequence rule along with the program rule to be applied in several places in order to simplify presentation. As a result, some detailed analysis is omitted, such as proving supports are disjoint in order to use the frame rule.

$$\{list(i) \wedge list(j) \wedge Sp(list(i)) \cap Sp(list(j)) = \emptyset \wedge i \neq nil\} \qquad \text{(consequence rule)}$$

[3] The acronym MW is a shout-out to the Magic-Wand operator, as these serve a similar function, except that they are definable in FL itself.

$$\{list(i) \wedge list(j) \wedge Sp(list(i)) \cap Sp(list(j)) = \emptyset \wedge i \neq nil \wedge i \notin Sp(list(j))\}$$
(consequence rule: unfolding list definition)

$$\{\exists k' : k' = next(i).\ list(k') \wedge i \notin Sp(list(k')) \wedge list(j) \wedge i \notin Sp(list(j)) \wedge Sp(list(k')) \cap Sp(list(j)) = \emptyset\}$$ (consequence rule)

$$\{\exists k' : k' = next(i).\ next(i) = next(i) \wedge list(k') \wedge i \notin Sp(list(k')) \wedge list(j) \wedge i \notin Sp(list(j)) \wedge Sp(list(k')) \cap Sp(list(j)) = \emptyset\}$$

```
k := i.next ;
```
(consequence rule, lookup-G rule)

$$\{next(i) = next(i) \wedge list(k) \wedge i \notin Sp(list(k)) \wedge list(j) \wedge i \notin Sp(list(j)) \wedge Sp(list(k)) \cap Sp(list(j)) = \emptyset\}$$

```
i.next := j ;
```
(mutation rule, frame rule)

$$\{next(i) = j \wedge list(k) \wedge i \notin Sp(list(k)) \wedge list(j) \wedge i \notin Sp(list(j)) \wedge Sp(list(k)) \cap Sp(list(j)) = \emptyset\}$$ (consequence rule)

$$\{list(k) \wedge next(i) = j \wedge i \notin Sp(list(j)) \wedge list(j) \wedge Sp(list(k)) \cap Sp(list(j)) = \emptyset\}$$
(consequence rule: folding list definition)

$$\{list(k) \wedge list(i) \wedge Sp(list(k)) \cap Sp(list(i)) = \emptyset\}$$

```
j := i ; i := k
```
(assignment-G rule)

$$\{list(i) \wedge list(j) \wedge Sp(list(i)) \cap Sp(list(j)) = \emptyset\}$$

Armed with this, proving $j$ is a list after executing the full program above is a trivial application of the assignment, while, and consequence rules, which we omit for brevity.

Observe that in the above proof we were apply the frame rule because of the fact that $i$ belongs neither to $Sp(list(k))$ nor $Sp(list(j))$. This can be dispensed with easily using reasoning about first-order formulae with least-fixpoint definitions, techniques for which are discussed in Section 6.

Also note the invariant of the loop is precisely the intended meaning of $list(i) * list(j)$ in separation logic. In fact, as we will see in Section 6, we can define a *first-order* macro *Star* as $Star(\varphi, \psi) = \varphi \wedge \psi \wedge Sp(\varphi) \cap Sp(\psi) = \emptyset$. We can use this macro to represent disjoint supports in similar proofs.

These proofs demonstrate what proofs of actual programs look like in our program logic. They also show that frame logic and our program logic can prove many results similarly to traditional separation logic. And, by using the derived operator *Star*, very little even in terms of verbosity is sacrificed in gaining the flexibility of Frame Logic(please see Section 6 for a broader discussion of the ways in which Frame Logic differs from Separation Logic and in certain situations offers many advantages in stating and reasoning with specifications/invariants).

### 5 Expressing a Precise Separation Logic

In this section, we show that FL is expressive by capturing a fragment of separation logic in frame logic; the fragment is a syntactic fragment of separation logic that defines only *precise formulas*— formulas that can be satisfied in at most one heaplet for any store. The translation also shows that frame logic can naturally and compactly capture such separation logic formulas.

#### 5.1 A Precise Separation Logic

As discussed in Section 1, a crucial difference between separation logic and frame logic is that formulas in separation logic have uniquely determined supports/heaplets, while this is not true in separation logic. However, it is well known that in verification, determined heaplets are very natural (most uses of separation logic in fact are precise) and sometimes desirable. For instance, see [8] where precision is used crucially to give sound semantics to concurrent separation logic and [29] where precise formulas are proposed in verifying modular programs as imprecision causes ambiguity in function contracts.

We define a fragment of separation logic that defines precise formulas (more accurately, we handle a slightly larger class inductively: formulas that when satisfiable have unique minimal heaplets for any given store). The fragment we capture is similar to the notion of precise predicates seen in [29]:

**Definition 3.** *PSL Fragment:*

- *sf: formulas over the stack only (nothing dereferenced). Includes* $isatom?()$*,* $m(x) = y$ *for immutable* $m$*, true, background formulas, etc.*
- $x \xrightarrow{f} y$
- $ite(sf, \varphi_1, \varphi_2)$ *where* $sf$ *is from the first bullet*
- $\varphi_1 \wedge \varphi_2$ *and* $\varphi_1 * \varphi_2$
- $\mathcal{I}$ *where* $\mathcal{I}$ *contains all unary inductive definitions* $I$ *that have unique heaplets inductively (list, tree, etc.). In particular, the body* $\rho_I$ *of* $I$ *is a formula in the PSL fragment (*$\rho_I[I \hookleftarrow \varphi]$ *is in the PSL fragment provided* $\varphi$ *is in the PSL fragment). Additionally, for all* $x$*, if* $s, h \models I(x)$ *and* $s, h' \models I(x)$*, then* $h = h'$*.*[4]
- $\exists y.\ (x \xrightarrow{f} y) * \varphi_1$

Note that in the fragment negation and disjunction are disallowed, but mutually exclusive disjunction using *ite* is allowed. Existential quantification is only present when the topmost operator is a $*$ and where one of the formulas guards the quantified variable uniquely.

The semantics of this fragment follows the standard semantics of separation logic [12, 27, 28, 38], with the heaplet of $x \xrightarrow{f} y$ taken to be $\{x\}$. See Remark 1 in Section 3.2 for a discussion of a more accurate heaplet for $x \xrightarrow{f} y$ being the set containing the pair $(x, f)$, and how this can be modeled in the above semantics by using field-lookups using non-mutable pointers.

**Theorem 4 (Minimum Heap).** *For any formula* $\varphi$ *in the PSL fragment, if there is an* $s$ *and* $h$ *such that* $s, h \models \varphi$ *then there is a* $h_\varphi$ *such that* $s, h_\varphi \models \varphi$ *and for all* $h'$ *such that* $s, h' \models \varphi$*,* $h_\varphi \subseteq h'$*.*

[4] While we only assume unary inductive definitions here, we can easily generalize this to inductive definitions with multiple parameters.

#### 5.2 Translation to Frame Logic

For a separation logic store and heap $s, h$ (respectively), we define the corresponding interpretation $\mathcal{M}_{s,h}$ such that variables are interpreted according to $s$ and values of pointer functions on $dom(h)$ are interpreted according to $h$. For $\varphi$ in the PSL fragment, we first define a formula $P(\varphi)$, inductively, that captures whether $\varphi$ is precise. $\varphi$ is a precise formula iff, when it is satisfiable with a store $s$, there is exactly one $h$ such that $s, h \models \varphi$. The formula $P(\varphi)$ is in separation logic and will be used in the translation. To see why this formula is needed, consider the formula $\varphi_1 \wedge ite(sf, \varphi_2, \varphi_3)$. Assume that $\varphi_1$ is imprecise, $\varphi_2$ is precise, and $\varphi_3$ is imprecise. Under conditions where $sf$ is true, the heaplets for $\varphi_1$ and $\varphi_2$ must align. However, when $sf$ is false, the heaplets for $\varphi_1$ and $\varphi_3$ can be anything. Because we cannot initially know when $sf$ will be true or false, we need this separation logic formula $P(\varphi)$ that is true exactly when $\varphi$ is precise.

**Definition 4.** *Precision predicate $P$:*

- $P(sf) = \bot$ *and* $P(x \xrightarrow{f} y) = \top$
- $P(ite(sf, \varphi_1, \varphi_2)) = (sf \wedge P(\varphi_1)) \vee (\neg sf \wedge P(\varphi_2))$
- $P(\varphi_1 \wedge \varphi_2) = P(\varphi_1) \vee P(\varphi_2)$
- $P(\varphi_1 * \varphi_2) = P(\varphi_1) \wedge P(\varphi_2)$
- $P(I) = \top$ *where* $I \in \mathcal{I}$ *is an inductive predicate*
- $P(\exists y.\ (x \xrightarrow{f} y) * \varphi_1) = P(\varphi_1)$

Note that this definition captures precision within our fragment since stack formulae are imprecise and pointer formulae are precise. The argument for the rest of the cases follow by simple structural induction.

Now we define the translation $T$ inductively:

**Definition 5.** *Translation from PSL to Frame Logic:*

- $T(sf) = sf$ *and* $T(x \xrightarrow{f} y) = (f(x) = y)$
- $ite(sf, \varphi_1, \varphi_2) = ite(T(sf), T(\varphi_1), T(\varphi_2))$
- $T(\varphi_1 \wedge \varphi_2) = T(\varphi_1) \wedge T(\varphi_2) \wedge T(P(\varphi_1)) \implies Sp(T(\varphi_2)) \subseteq Sp(T(\varphi_1))$
  $\wedge\ T(P(\varphi_2)) \implies Sp(T(\varphi_1)) \subseteq Sp(T(\varphi_2))$
- $T(\varphi_1 * \varphi_2) = T(\varphi_1) \wedge T(\varphi_2) \wedge Sp(T(\varphi_1)) \cap Sp(T(\varphi_2)) = \emptyset$
- $T(I) = T(\rho_I)$ *where* $\rho_I$ *is the definition of the inductive predicate* $I$ *as in Section 3.*
- $T(\exists y.\ (x \xrightarrow{f} y) * \varphi_1) = \exists y : [f(x) = y].\ [T(\varphi_1) \wedge x \notin Sp(T(\varphi_1))]$

Finally, recall that any formula $\varphi$ in the PSL fragment has a unique minimal heap (Theorem 4). With this (and a few auxiliarly lemmas that can be found in the Technical Report [25]), we have the following theorem, which captures the correctness of the translation:

**Theorem 5.** *For any formula $\varphi$ in the PSL fragment, we have the following implications:*

$$s, h \models \varphi \implies \mathcal{M}_{s,h} \models T(\varphi)$$

$$\mathcal{M}_{s,h} \models T(\varphi) \implies s, h' \models \varphi \text{ where } h' \equiv \mathcal{M}_{s,h}(Sp(T(\varphi)))$$

*Here, $\mathcal{M}_{s,h}(Sp(T(\varphi)))$ is the interpretation of $Sp(T(\varphi))$ in the model $\mathcal{M}_{s,h}$. Note $h'$ is minimal and is equal to $h_\varphi$ as in Theorem 4.*

### 6 Discussion

*Comparison with Separation Logic.* The design of frame logic is, in many ways, inspired by the design choices of separation logic. Separation logic formulas implicitly hold on *tight* heaplets— models are defined on pairs $(s, h)$, where $s$ is a store (an interpretation of variables) and $h$ is a heaplet that defines a subset of the heap as the domain for functions/pointers. In Frame Logic, we choose to not define satisfiability with respect to heaplets but define it with respect to the entire heap. However, we give access to the implicitly defined heaplet using the operator *Sp*, and give a logic over *sets* to talk about supports. The separating conjunction operation $*$ can then be expressed using normal conjunction and a constraint that says that the support of formulae are disjoint.

We do not allow formulas to have *multiple* supports, which is crucial as *Sp* is a function, and this roughly corresponds to *precise* fragments of separation logic. Precise fragments of separation logic have already been proposed and accepted in the separation logic literature for giving robust handling of modular functions, concurrency, etc. [8, 29]. Section 5 details a translation of a precise fragment of separation logic (with $*$ but not magic wand) to frame logic that shows the natural connection between precise formulas in separation logic and frame logic.

Frame logic, through the support operator, facilitates local reasoning much in the same way as separation logic does, and the frame rule in frame logic supports frame reasoning in a similar way as separation logic. The key difference between frame logic and separation logic is the adherence to a first-order logic (with recursive definitions), both in terms of syntax and expressiveness.

First and foremost, in separation logic, the magic wand is needed to express the weakest precondition [38]. Consider for example computing the weakest precondition of the formula $list(x)$ with respect to the code $y.n := z$. The weakest precondition should essentially describe the (tight) heaplets such that changing the $n$ pointer from $y$ to $z$ results in $x$ pointing to a list. In separation logic, this is expressed typically (see [38]) using magic wand as $(y \xrightarrow{n} z) \; -\!\!* \; (list(x))$. However, the magic wand operator is inherently a *second-order* property. The formula $\alpha -\!\!* \beta$ holds on a heaplet $h$ if for any *disjoint* heaplet that satisfies $\alpha$, $\beta$ will hold on the conjoined heaplet. Expressing this property (for arbitrary $\alpha$, whose heaplet can be *unbounded*) requires quantifying over unbounded heaplets satisfying $\alpha$, which is not first order expressible.

In frame logic, we instead rewrite the recursive definition $list(\cdot)$ to a new one $list'(\cdot)$ that captures whether $x$ points to a list, assuming that $n(y) = z$ (see Section 4.4). This property continues to be expressible in frame logic and can be converted to first-order logic with recursive definitions (see Section 3.5). Note that we are exploiting the fact that there is only a bounded amount of change to the heap in straight-line programs in order to express this in FL.

Let us turn to expressiveness and compactness. In separation logic, separation of structures is expressed using $*$, and in frame logic, such a separation is expressed using conjunction and an additional constraint that says that the supports of the two formulas are disjoint. A precise separation logic formula of the form $\alpha_1 * \alpha_2 * \ldots \alpha_n$ is compact and would get translated to a much larger formula in frame logic as it would have to state that the supports of each pair of formulas is disjoint. We believe this can be tamed using macros ($Star(\alpha, \beta) = \alpha \wedge \beta \wedge Sp(\alpha) \cap Sp(\beta) = \emptyset$).

There are, however, several situations where frame logic leads to more compact and natural formulations. For instance, consider expressing the property that $x$ and $y$ point to lists, which may or may not overlap. In Frame Logic, we simply write $list(x) \wedge list(y)$. The support of this formula is the union of the supports of the two lists. In separation logic, we cannot use $*$ to write this compactly (while capturing the tightest heaplet). Note that the formula $(list(x) * true) \wedge (list(y) * true)$ is *not* equivalent, as it is true in heaplets that are larger than the set of locations of the two lists. The simplest formulation we know is to write a recursive definition $lseg(u, v)$ for list segments from $u$ to $v$ and use quantification: $(\exists z.\ lseg(x, z) * lseg(y, z) * list(z)) \vee (list(x) * list(y))$ where the definition of $lseg$ is the following: $lseg(u, v) \equiv (u = v \wedge emp) \vee (\exists w.\ u \to w\ *\ lseg(w, v))$.

If we wanted to say $x_1, \ldots, x_n$ all point to lists, that may or may not overlap, then in FL we can say $list(x_1) \wedge list(x_2) \wedge \ldots \wedge list(x_n)$. However, in separation logic, the simplest way seems to be to write using $lseg$ and a linear number of quantified variables and an exponentially-sized formula. Now consider the property saying $x_1, \ldots, x_n$ all point to binary trees, with pointers *left* and *right*, and that can overlap arbitrarily. We can write it in FL as $tree(x_1) \wedge \ldots \wedge tree(x_n)$, while a formula in (first-order) separation logic that expresses this property seems very complex.

In summary, we believe that frame logic is a logic that supports frame reasoning built on the same principles as separation logic, but is still translatable to first-order logic (avoiding the magic wand), and makes different choices for syntax/semantics that lead to expressing certain properties more naturally and compactly, and others more verbosely.

*Reasoning with Frame Logic using First-Order Reasoning Mechanisms.* An advantage of the adherence of frame logic to being translatable to a first-order logic with recursive definitions is the power to reason with it using first-order theorem proving techniques. While we do not present tools for reasoning in this paper, we note that there are several reasoning schemes that can readily handle first-order logic with recursive definitions.

The theory of dynamic frames [18] has been proposed for frame reasoning for heap manipulating programs and has been adopted in verification engines like Dafny [21] that provide automated reasoning. A key aspect of dynamic frames is the notion of regions, which are subsets of locations that can be used to define subsets of the heap that change or do not change when a piece of code is executed. Program logics such as region logic have been proposed for object-oriented programs using such regions [1–3]. The supports of formulas in frame logic are also used to express such regions, but the key difference is that the definition of regions is given *implicitly* using supports of formulas, as opposed to explicitly defining them. Separation logic also defines regions implicitly, and in fact, the work on implicit dynamic frames [31, 39] provides translations from separation logic to regions for reasoning using dynamic frames.

Reasoning with regions using set theory in a first-order logic with recursive definitions has been explored by many works to support automated reasoning. Tools like Vampire [20] for first-order logic have been extended in recent work to handle algebraic datatypes [19]; many data-structures in practice can be modeled as algebraic datatypes and the schemes proposed in [19] are powerful tools to reason with them using first-order theorem provers.

A second class of tools are those proposed in the work on natural proofs [23, 32, 37]. Natural proofs explicitly work with first order logic with recursive definitions (FO-RD), implementing validity through a process of unfolding recursive definitions, uninterpreted abstractions, and proving inductive lemmas using induction schemes. Natural proofs are currently used primarily to reason with separation logic by first translating verification conditions arising from Hoare triples with separation logic specifications (without magic wand) to first-order logic with recursive definitions. Frame logic reasoning can also be done in a very similar way by translating it first to FO-RD.

The work in [23] considers natural proofs and quantifier instantiation heuristics for FO-RD (using a similar setup of foreground sort for locations and background sorts), and the work identifies a fragment of FO-RD (called safe fragment) for which this reasoning is *complete* (in the sense that a formula is detected as unsatisfiable by quantifier instantiation iff it is unsatisfiable with the inductive definitions interpreted as fixpoints and not least fixpoints). Since FL can be translated to FO-RD, it is possible to deal with FL using the techniques of [23]. The conditions for the safe fragment of FO-RD are that the quantifiers over the foreground elements are the outermost ones, and that terms of foreground type do not contain variables of any background type. As argued in [23], these restrictions are typically satisfied in heap logic reasoning applications.

### 7 Related Work

The frame problem [13] is an important problem in many different domains of research. In the broadest form, it concerns representing and reasoning about the effects of a local action without requiring explicit reasoning regarding static changes to the global scope. For example, in artificial intelligence one wants a logic that can seamlessly state that if a door is opened in a lit room, the lights continue to stay switched on. This issue is present in the domain of verification as well, specifically with heap-manipulating programs.

There are many solutions that have been proposed to this problem. The most prominent proposal in the verification context is separation logic [12, 27, 28, 38], which we discussed in detail in the previous section.

In contrast to separation logic, the work on Dynamic Frames [17, 18] and similarly inspired approaches such as Region Logic [1–3] allow methods to explicitly specify the portion of the support that may be modified. This allows fine-grained control over the modifiable section, and avoids special symbols like

$*$ and $-*$. However, explicitly writing out frame annotations can become verbose and tedious.

The work on Implicit Dynamic Frames [22, 39, 40] bridges the worlds of separation logic (without magic wand) and dynamic frames— it uses separation logic and fractional permissions to implicitly define frames (reducing annotation burden), allows annotations to access these frames, and translates them into set regions for first-order reasoning. Our work is similar in that frame logic also implicitly defines regions and gives annotations access to these regions, and can be easily translated to pure FO-RD for first-order reasoning.

One distinction with separation logic involves the non-unique heaplets in separation logic and the unique heaplets in frame logic. Determined heaplets have been used [29, 32, 37] as they are more amenable to automated reasoning. In particular a separation logic fragment with determined heaplets known as precise predicates is defined in [29], which we capture using frame logic in Section 5.

There is also a rich literature on reasoning with these heap logics for program verification. Decidability is an important dimension and there is a lot of work on decidable logics for heaps with separation logic specifications [4–6, 11, 26, 33]. The work based on EPR (Effectively Propositional Reasoning) for specifying heap properties [14–16] provides decidability, as does some of the work that translates separation logic specifications into classical logic [34].

Finally, translating separation logic into classical logics and reasoning with them is another solution pursued in a lot of recent efforts [10, 23, 24, 32, 32, 34–37, 41]. Other techniques including recent work on cyclic proofs [9, 42] use heuristics for reasoning about recursive definitions.

### 8 Conclusions

Our main contribution is to propose *Frame Logic*, a classical first-order logic endowed with an explicit operator that recovers the implicit supports of formulas and supports frame reasoning. we have argued its expressive by capturing several properties of data-structures naturally and succinctly, and by showing that it can express a precise fragment of separation logic. The program logic built using frame logic supports local heap reasoning, frame reasoning, and weakest tightest preconditions across loop-free programs.

We believe that frame logic is an attractive alternative to separation logic, built using similar principles as separation logic while staying within the first-order logic world. The first-order nature of the logic makes it potentially amenable to easier automated reasoning.

A practical realization of a tool for verifying programs in a standard programming language with frame logic annotations by marrying it with existing automated techniques and tools for first-order logic (in particular [19, 24, 32, 37, 41]), is the most compelling future work.

**Acknowledgements:** We thank ESOP'20 reviewers for their comments that helped improve this paper. This work is based upon research supported by the National Science Foundation under Grant NSF CCF 1527395.
